# SiamFDA: feature dynamic activation siamese network for visual tracking

**DOI:** 10.1038/s41598-024-55487-7

**Published:** 2024-02-27

**Authors:** Jialiang Gu, Ying She, Yi Yang

**Affiliations:** Computer Science and Engineering, Sun Yat-sen University, Guangzhou, 510000 China

**Keywords:** Computer science, Software

## Abstract

In this paper, we present a novel anchor-free visual tracking framework, referred to as feature dynamic activation siamese network (SiamFDA), which addresses the issue of ignoring global spatial information in current Siamese network-based tracking algorithms. Our approach captures long-range dependencies between distant pixels in space, which enables robustness to unreliable regions. Additionally, we introduce a hierarchical feature selector that adaptively activates features at different layers, and an adaptive sample label assignment method to further improve tracking performance. Our extensive evaluations on six benchmark datasets, including VOT-2018, VOT-2019, GOT10k, LaSOT, OTB-2015, and OTB-2013, demonstrate that SiamFDA outperforms several state-of-the-art trackers in various challenging scenarios, with a real-time frame rate of 40 frames per second.

## Introduction

Visual tracking is a fundamental task in computer vision, with various practical applications in the real world such as video surveillance, human–machine interaction and biomedical image analysis. Generally, given the initial state of a target, we are expected to predict its motion trajectory in subsequent frames. Though many efforts have been done recently, visual tracking still needs to cope with scale variation, appearance deformation, background clutter and so on.

**Figure 1:**
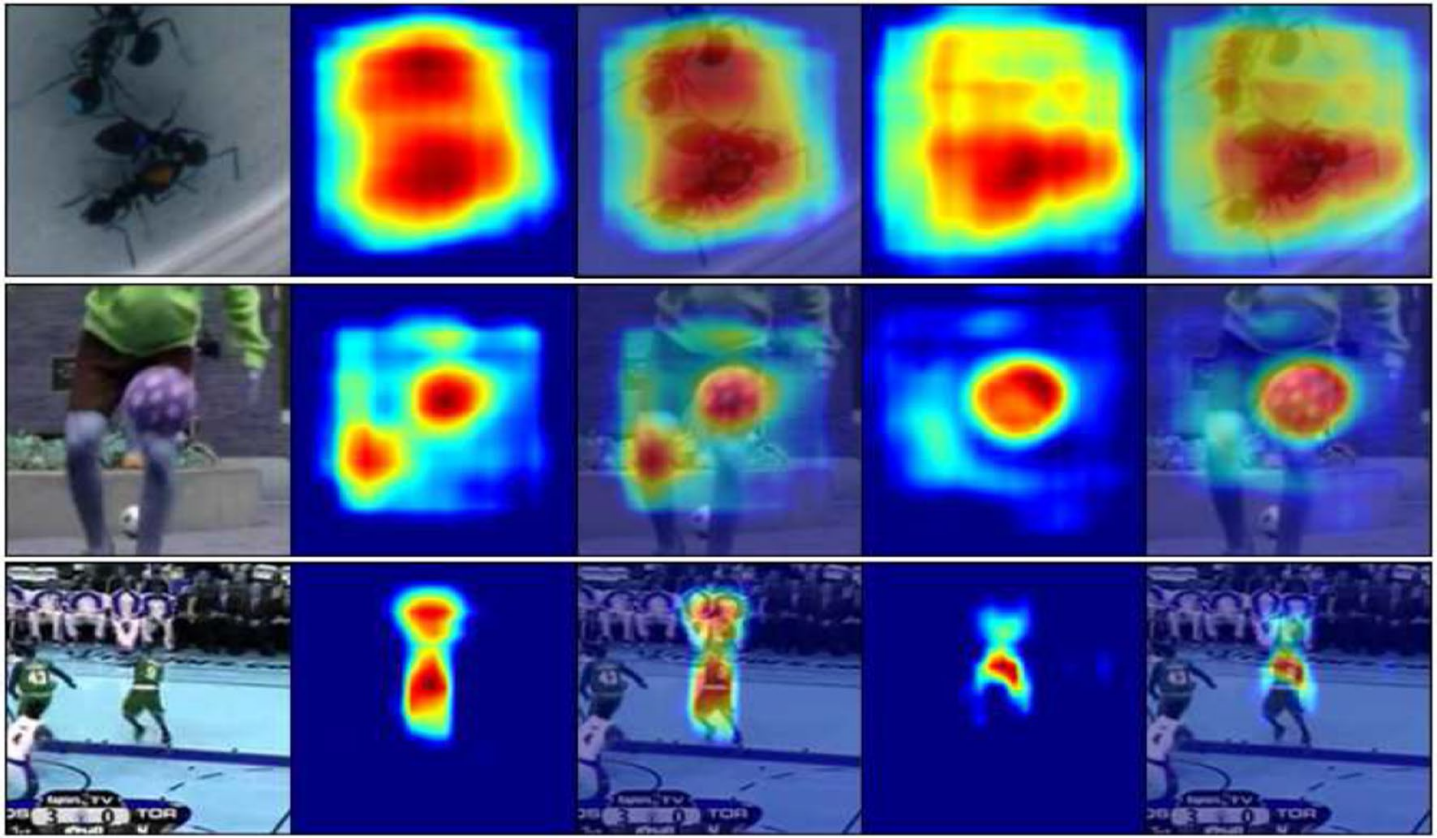
Visualization of attention maps (heatmaps) of SiamBAN (column 2 and 3) and our proposed SiamFDA (column 4 and 5) on three challenging video sequences, from which, we can see that SiamFDA can effectively identify ambiguous patches and enables our model to be robust to the unreliable regions.

Recently, tracking algorithms based on the Siamese network^1,2^ have attracted great attention because of their balanced accuracy and speed. The pioneering works SiamFC^1^ simply matches the initial patch of the target in the first frame with candidates in subsequent frames and returns the most similar patch by a learned matching function. SiamRPN^2^ introduces the region proposal network to discard traditional multi-scale tests, which inevitably introduces many anchor related hyper-parameters that require carefully tuning and heavy computational burdens. To solve these problems, SiamBAN^3^ introduces an anchor-free tracker, which directly regresses the positions of the target in a video frame. Although above methods have obtained excellent performance on visual object tracking, they merely focus on the local characteristics of the target, and inevitably ignores the intrinsic structural information within the global region. These long-range features are particularly suitable for specific constraints of set prediction^4^ such as background clutter and other challenges. Therefore, as Fig. 1 shown, SiamBAN^3^ cannot identify the target ant from similar objects in the first sequence, and even cannot discriminate different objects such as between the knee and the football. Recently, non-local network (NLNet)^5^ is proposed to model the long-range dependencies via self-attention mechanism^6^. Intuitively, a NL block compute the response at a position as a weighted sum of the features at all positions in the input feature map, to attain an attention map. Then the input features are aggregated with the important weights defined by the above attention map, thus allowing distant pixels to contribute to the filtered response at a local location. However, For an image, different query positions get almost the same global context information through the non-local structure^7^. Moreover, NL block has to compute the pixel-level pairwise relations among all positions, which results in a heavy computational load.

**Figure 2:**
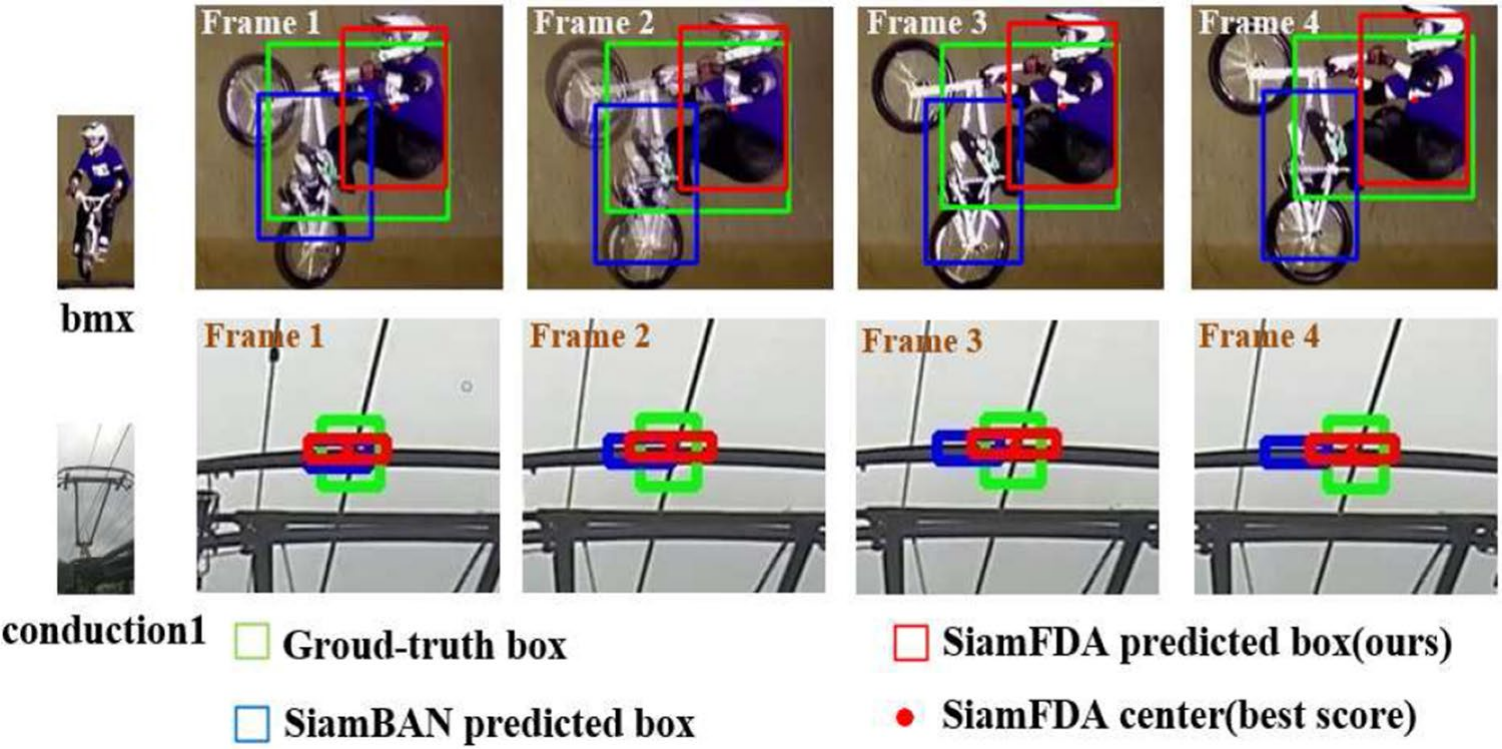
Qualitative comparison of our SiamFDA with SiamBAN on VOT-2018. Frames 1, 2, 3, and 4, each representing a consecutive frame in the tracking process. Observed from the visualization results, our tracker is better than the baseline in terms of accurate tracking.

In this work, we propose a simple yet effective anchor-free visual tracking framework named feature dynamic activation siamese network (SiamFDA), which consists of a Siamese network backbone for feature extraction and a feature dynamic activation (FDA) subnetwork for accurate target location estimation as well as bounding box prediction. Specifically, we design a novel FDA block for efficiently modeling long-range dependencies of the target and its modeling framework can be abstracted into three steps: (1) context modeling module obtains position-independent context information as attention weights to make the tracking model focus on crucial regions. (2) Transform module further strengthens the representation power of the meaningful contextual information and captures the channel-wise interdependencies at the same time. (3) Fusion module merges the original input feature with global context features to improve discriminability. Besides, to fuse fine-grained information and abstract semantic information of features adaptively, we introduce a squeeze-and-excitation (SE) block^8^, which makes up for the lack of channel attention. Furthermore, on the observation that when the target aspect ratio is close to 1, the number of positive samples captured by an ellipse is less than a circle, we modify the original label assignment method to add more reliable samples, thus improving the tracking accuracy to some extent. Figure 1 displays that compared with SiamBAN^3^, our SiamFDA pays more attention to the tracking target without being misled by similar objects and the background. For example, in the third sequence, our SiamFDA would focus more on the player’s jersey number instead of other places, which is more consistent with human perception. When we look at the fast-moving players on the court, the jersey numbers can help us quickly determine the identity of the player. In Fig. 2, we provide a qualitative comparison between our SiamFDA and SiamBAN on the VOT-2018 dataset. It is evident from the visualization results that our tracker outperforms the baseline (SiamBAN) in terms of precise tracking.

The main contributions of our work can be summarized as:

- We propose a simple yet effective anchor-free Siamese network SiamFDA to accurately estimate scale variation and aspect-ratio changes, thus boosting the generalization ability of the tracker.
- We design a novel FDA block which encodes rich global context information into the target representation along the spatial dimension. This block activates reliable patches, and enables our model to be robust to the unreliable regions during tracking. Furthermore, we adopt the SE block as a hierarchical feature selector in the classification and regression branches, which further maximizes the discriminative abilities via exploiting the inter-channel relationship.
- We introduce an adaptive sample label assignment method to add more reliable positive samples, thus improving the tracking performance.
- The effectiveness of SiamFDA is verified on six datasets, and the results demonstrate that SiamFDA is very promising for various challenging scenarios compared with several state-of-the-art(SOTA) trackers, with real-time performance of 40 fps.

## Related work

### Visual tracking

Recently, the proposal of Siamese network is a pioneering work in visual tracking community due to its end-to-end training capabilities and high efficiency. SiamFC^1^ presents a real-time tracking algorithm that utilizes a novel fully-convolutional Siamese network, trained end-to-end. SiamRPN^2^ introduces a region proposal network for precise bounding box regression. Building upon this, SiamRPN++^10^ architecture for improved performance. Although these anchor-based methods effectively address scale variation and aspect ratio changes, they introduce numerous additional hyper-parameters that necessitate careful tuning and impose significant computational burdens. Furthermore, the anchor setting is not in line with the spirit of generic visual tracking, as it requires pre-defined hyper-parameters to describe the shape. Therefore, SiamFC++^11^ introduces a set of guidelines that include the decomposition of classification and state estimation, non-ambiguous scoring, being prior knowledge-free, and estimation quality assessment. SiamBAN^3^ propose a simple yet effective visual tracking framework by exploiting the expressive power of the fully convolutional network. With the emergence of Transformer architectures, their significant advantages in handling complex sequential data have increasingly captured the attention of researchers in the academic field. Despite this, Transformer-based trackers^12–18^ face significant challenges in practical applications, particularly due to their higher computational burden, which limits their feasibility in real-time tracking scenarios. In contrast, while CNN-based trackers may lag behind Transformer-based models in certain performance metrics, their lower computational complexity makes them more advantageous in scenarios requiring quick response times.

Similar to SiamBAN^3^, we design an anchor-free Siamese network, which avoids hyper-parameters associated with the candidate boxes and makes the tracker more flexible and general.

### Long-range dependency modeling

Recently, many new approaches focusing on long-range dependency modeling have emerged in object classification and detection. To model the pairwise relation, NLNet^5^ computes the response at a position as a weight sum of the features at all positions. GCNet^7^ has found that the global contexts modeled by NLNet^5^ are almost the same for different positions within an image. Therefore, GCNet^7^ creates a simplified network based on a query-independent formulation, which maintains the accuracy of NLNet^5^ but with significantly less computation. To model the query-independency global context, SENet^8^ focuses on the channel relationship and adaptively recalibrates channel-wise feature responses. CBAM^19^ exploits both spatial and channel-wise attention based on an efficient architecture. Particularly, the recent advance of tracking approaches has achieved great success by integrating attention mechanisms. SiamAttn^20^ learns strong context information and aggregates rich contextual inter-dependencies between two branches of Siamese network, via deformable self-attention and cross-attention jointly.

In our paper, we introduce a novel FDA block designed to effectively model long-range dependencies, addressing the NL block’s inherent limitations. This approach enables our model to adaptively focus on reliable regions across the spatial dimension. The SE block is further exploited to determine the effectiveness of each output channel.

**Figure 3:**
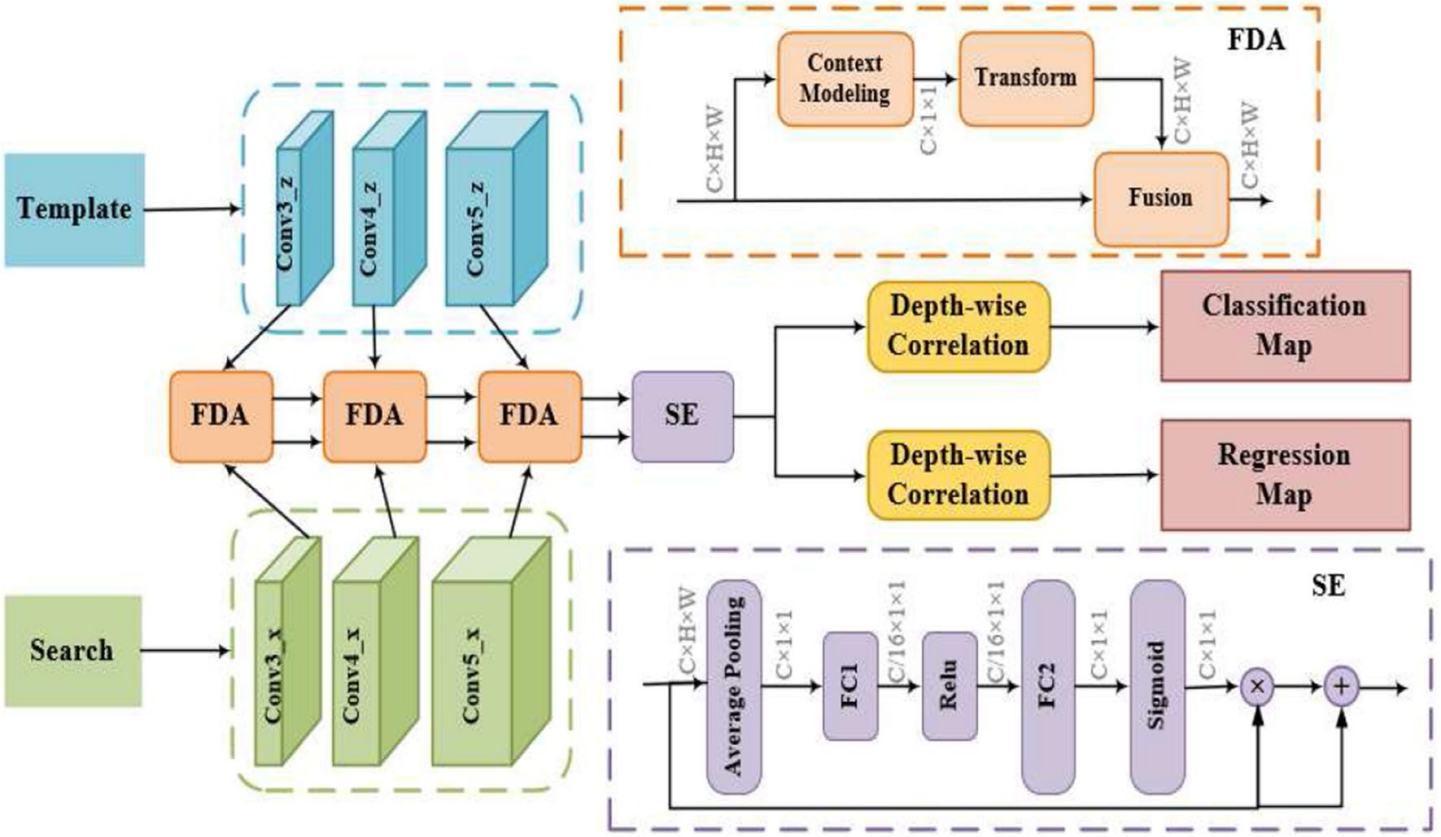
Overview of the proposed SiamFDA architecture. The top branch is the template branch which encodes the appearance information of the target, and the bottom branch is the search branch. $Conv3\_z$, $Conv4\_z$ and $Conv5\_z$ represent the feature maps of the template branch while $Conv3\_x$, $Conv4\_x$ and $Conv5\_x$ represents the feature maps of the search branch. The features of each stages from the Siamese network backbone are extracted and then modulated by three FDA blocks, which generates global context features and feeds them into a SE block to further exploit the channel attention. The network finally outputs a 2*D* classification map and a 4*D* regression map.

## SiamFDA framework

As displayed in Fig. 3, the proposed SiamFDA consists of a Siamese network backbone for feature extraction and a FDA subnetwork for accurate target location estimation as well as bounding box prediction. Specifically, the Siamese network backbone encodes the appearance information of the template image and the search image. The FDA subnetwork includes a classification branch and a regression branch, which considers the spatial layouts of the target and models the query-independency global context via three novel FDA blocks. Besides, a SE block is introduced to further amplify the discriminative ability along the channel dimension.

### Revisiting Siamese network backbone

The Siamese network-based trackers view visual tracking as a cross-correlation problem and learn a tracking similarity map from a fully-convolutional network, which compares a template image Z against a search image X of the same size and returns a high score if the two images depict the same object and a low score otherwise. We use the initial appearance feature of the target as the template and a larger crop centered on the last estimated position of the target as the input of the search branch. These two branches share parameters in the Siamese backbone so that the two patches are implicitly encoded by the same transformation which is suitable for the subsequent network. We use the modified ResNet-50^3^ pretrained from ImageNet^21^ as the backbone. The down-sampling operations from the last two convolution blocks are removed to reserve detailed spatial information and thus perform dense prediction. Besides, atrous convolutions with different atrous rates are adopted to improve the receptive field.

### Feature dynamic activation subnetwork

**Figure 4:**
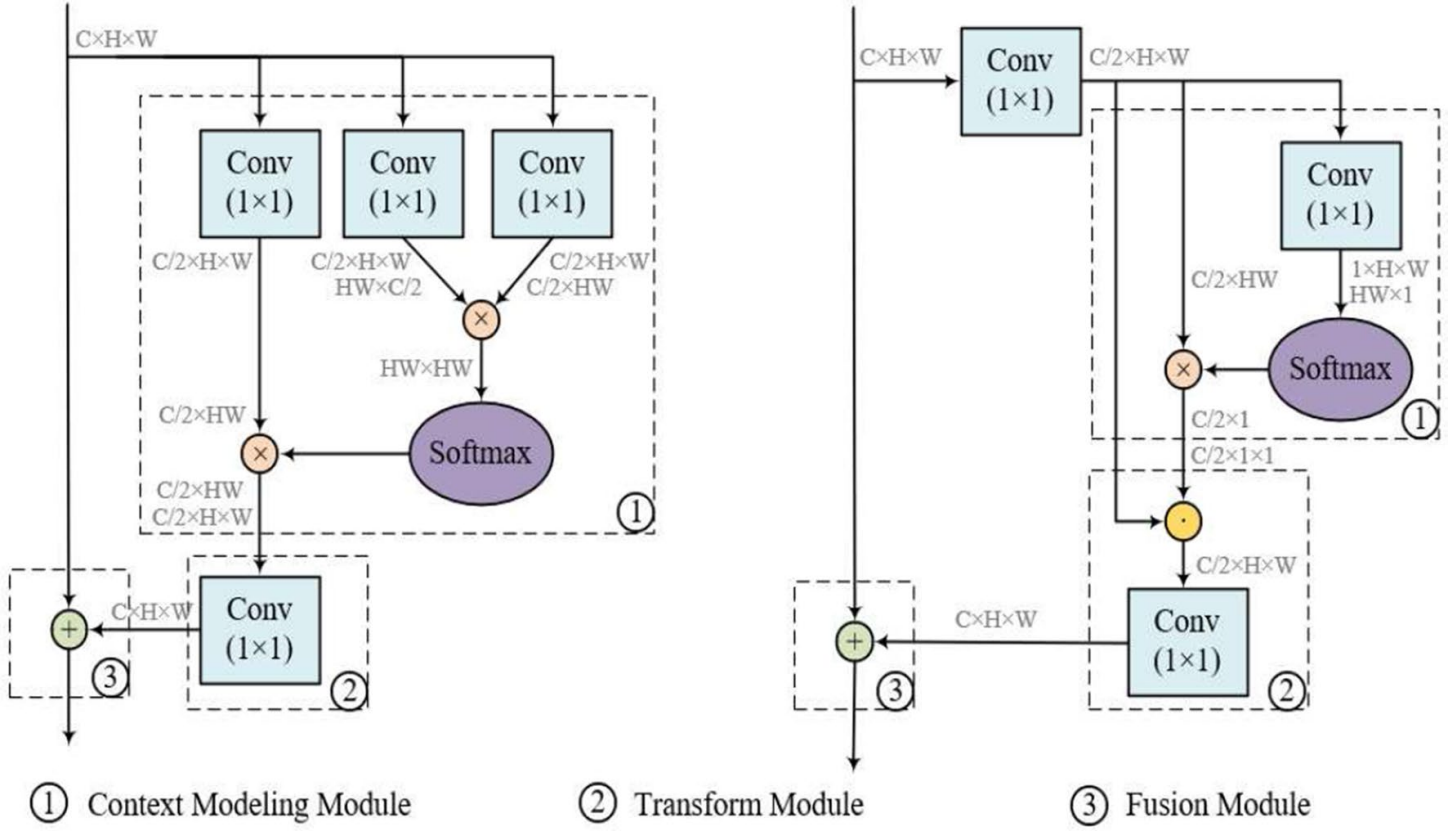
Architecture of the NL block (left) and our FDA block (right), both of which contain three modules: context modeling module, transform module and fusion module. The feature maps are displayed as feature dimensions, e.g., $C\times H \times W$ denotes that a feature map with channel number *C*, height *H* and width *W*. $\otimes$ denotes matrix multiplication, $\bigodot$ denotes element-wise multiplication and $\bigoplus$ denotes element-wise addition. The blue boxes denote $1\times 1$ convolution and the purple ellipses denote the softmax operation.

FDA subnetwork consists of a classification branch and a regression branch, which captures long-range dependencies of the target via three novel FDA blocks. As illustrated in Fig. 4, our FDA block contains three modules: context modeling module, transform module and fusion module. Specifically, as different instantiations achieve comparable performance^5^, we adopt embedded Gaussian as the basic NL block to compute similarity in an embedding space. Suppose the input features are *X*, with shapes of $N_{p}=C\times H \times W$. *H* represents the height of the target, *W* denotes the width and *C* denotes the channel.

#### Context modeling module

Based on the observation that the attention maps for different positions are almost the same in the NLNet^5^, we replace the pixel-level pairwise operation with a $1\times 1$ convolution $W_{c}$, and obtain a position-independent attention map via a softmax function. Then these attention weights are aggregated with the input features by matrix multiplication, to recalibrate the importance of different spatial positions. Thus, the context modeling procedure can be formulated as

1 $$\begin{aligned} {\bar{X}}_i=\sum _{j=1}^{N_p}\frac{\exp ({W_c}{X_j})}{\sum _{m=1}^{N_p}{\exp (W_cX_m)}}X_j, \end{aligned}$$

where *i* denotes the index of query positions and *j* enumerates all possible position.

#### Transform module

To further strengthen the representation power of global context features, we aggregate the global context features to each position of the input feature via element-wise multiplication, and adopt a $1\times 1$ convolution $W_t$ to capture channel-wise dependencies, as $W_t({\bar{X}}_i\cdot X_j)$.

#### Fusion module

We broadcast the simple element-wise addition for final feature fusion. Besides, a subsampling trick via a $1\times 1$ convolution $W_s$ is used before context modeling module to further lower computation, as ${\hat{X}}_j= W_sX_j$ and ${\hat{X}}_m=W_sX_m$. Thus, the overall procedure can be expressed as

2 $$\begin{aligned} F^x=X_i+W_t\sum _{j=1}^{N_p}\frac{\exp (W_c{\hat{X}}_j)}{\sum _{m=1}^{N_p}{\exp (W_c{\hat{X}}_m)}}{\hat{X}}_j\cdot {\hat{X}}_j. \end{aligned}$$

In the paper, the FDA block is not inserted between features of different layers of the backbone, but acts directly on the output of layer 3–5, respectively. This not only effectively utilizes the global context feature information of different layers, but also avoids the false guidance of low-level features to high-level feature extraction. The final output can be attained by the concatenation operation.

Considering that FDA blocks mainly pay attention to the global spatial information which decides ‘where’ to focus, and miss the complementary channel attention which decides ‘what’ to focus, a SE block^8^ is introduced and placed in a sequential manner. The SE block serves as a hierarchical feature selector which directly selects features that are more conductive to identifying the current target and amplifies their discriminative abilities, leading to more accurate tracking. Specifically, the concatenated features from three FDA blocks are fed into a SE block, and are decoupled according to corresponding layers. For convenience, the decoupled feature of the template branch and the search branch is simply denoted as $F_{se}^z$ and $F_{se}^x$, respectively. Then, we copy $F_{se}^z$ and $F_{se}^x$ of each layer to the classification branch and the regression branch, denoted as $[F_{se}^z]_{cls}$, $[F_{se}^z]_{reg}$ and $[F_{se}^x]_{cls}$, $[F_{se}^x]_{reg}$. Each branch combines the feature maps via a depth-wise cross-correlation layer:

3 $$\begin{aligned} P_{cls}= & {} [F_{se}^z]_{cls}*[F_{se}^x]_{cls}, \end{aligned}$$

4 $$\begin{aligned} P_{reg}= & {} [F_{se}^z]_{reg}*[F_{se}^x]_{reg}, \end{aligned}$$

where $*$ represents the convolutional operation, $P_{cls}$ and $P_{reg}$ denote the classification and the regression map, respectively. Finally, the classification maps and the regression maps from different layers are fused independently, and the corresponding weights are optimized through training. Specifically, each location (*i*, *j*) on the classification map is considered as a positive sample if its corresponding position$(\lfloor \frac{w_{im}}{2}\rfloor +(i-\left\lfloor \frac{w_{im}}{2}\right\rfloor ) \times {s},\lfloor \frac{h_{im}}{2}\rfloor +(j-\left\lfloor \frac{h_{im}}{2}\right\rfloor ) \times {s})$ on the input image falls within the ground-truth bounding box, and a negative sample otherwise. Here, $w_{im}$ and $h_{im}$ represent the width and the height of the input image, and *s* denotes the total stride of the network. For each location (*i*, *j*) on the regression map, we estimate a 4*D* vector at each spatial location of the feature map. The 4*D* vector represents the relative offsets from the four sides of a bounding box to the center location.

**Figure 5:**
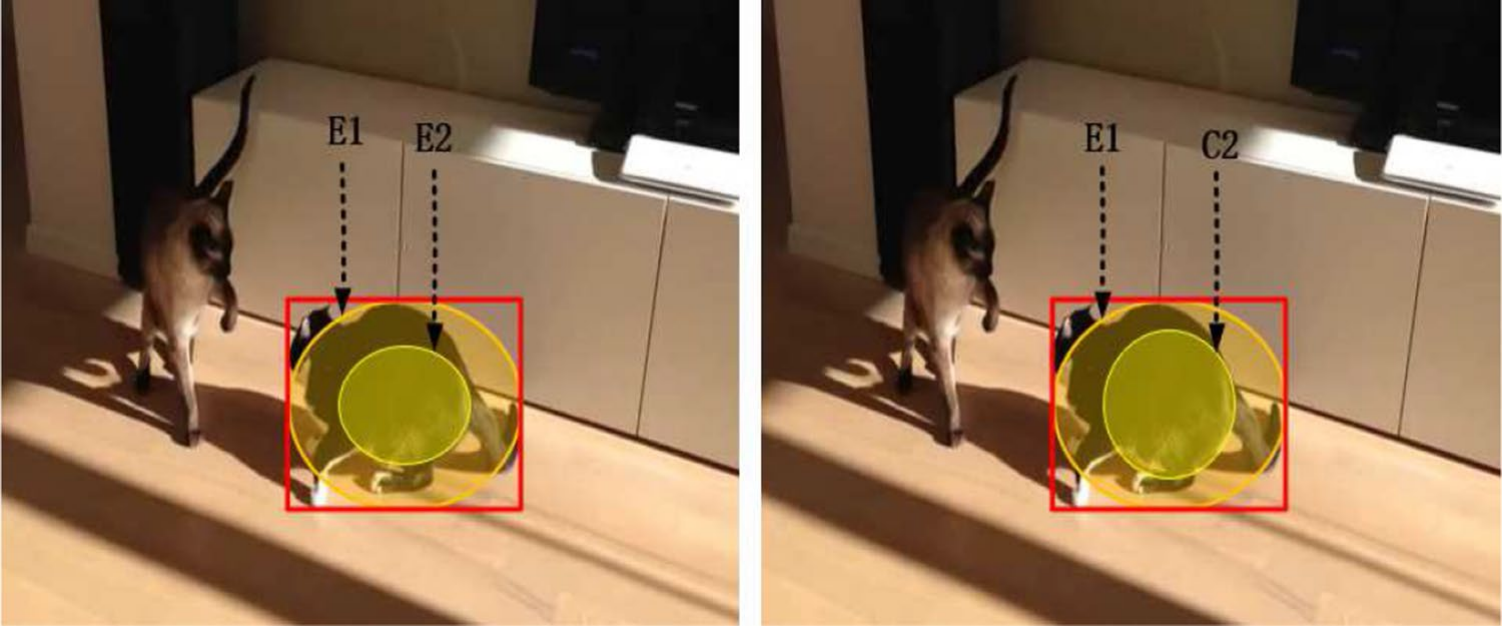
The sample label assignment methods of SiamBAN and SiamFDA. *E*1 denotes ellipse *E*1, which is the border for negative labels, *E*2 and *C*2 denote ellipse *E*2 and circle *C*2, which are the border for positive labels.

### Ground-truth and Loss

As illustrated in SiamBAN^3^, the sample label assignment is important for the tracking performance, which is usually ignored by most Siamese network-based trackers. SiamBAN^3^ adopts two ellipses to define both negative labels and positive labels. However, as Fig. 5 shown, we find that if the target aspect ratio is close to 1, which means that the target shape approximates a circle, the number of positive samples contained in the ellipse *E*2 is less than the circle *C*2. Therefore, to add more reliable positive samples, we preserve the setting for negative labels and modify for positive labels. Specifically, following the definitions^3^, the width, height, top-left corner, center point and bottom-right corner of the ground-truth bounding box are represented by $g_w$, $g_h$, $(g_{x1},g_{y1})$, $(g_{xc},g_{yc})$ and $(g_{x2}, g_{y2})$, respectively. Then the border for negative labels can be formulated as

5 $$\begin{aligned} E1:\frac{{(p_i-g_{xc})}^2}{{(\frac{g_w}{2})}^2}+\frac{{(p_j-g_{yc})}^2}{{(\frac{g_h}{2})}^2}=1, \end{aligned}$$

where $(p_i,p_j)$ denotes the location of the feature maps. The border for positive labels can be formulated as

6 $$\begin{aligned} E2:\frac{{(p_i-g_{xc})}^2}{{(\frac{g_w}{4})}^2}+\frac{{(p_j-g_{yc})}^2}{{(\frac{g_h}{4})}^2}=1, \end{aligned}$$

when $min{\left( g_w,g_h\right) }<0.25*max\left( g_w,g_h\right)$, which represents the target shape is close to a long rectangle. Under this circumstance, the area of the ellipse with $\frac{g_w}{4}$,$\frac{g_h}{4}$ as the axes length is larger than the area of the circle with $\min {\left( \frac{g_w}{2},\frac{g_h}{2}\right) }$ as the radius.

7 $$\begin{aligned} C2:\frac{{(p_i-g_{xc})}^2}{r^2}+\frac{{(p_j-g_{yc})}^2}{r^2}=1, \end{aligned}$$

when $r=min{\left( \frac{g_w}{2},\frac{g_h}{2}\right) }$ and $min{\left( g_w,g_h\right) }\ge 0.25*max\left( g_w,g_h\right)$, which represents the target shape is close to a square and the area of a circle is larger than an ellipse.

Therefore, the location $(p_i,p_j)$ is assigned with a positive label if falling within *E*2/*C*2, while a negative label if falling outside *E*1. The position falls between *E*2/*C*2 and *E*1 would be ignored. It should be noticed that only the location with a positive label would be used for bounding box regression. Finally, the multi-task loss function is minimized as

8 $$\begin{aligned} L=\lambda _1L_{cls}+\lambda _2L_{reg}, \end{aligned}$$

where $L_{cls}$ is the focal loss for the classification result, $L_{reg}$ is the intersection over union (IoU) loss for the regression result. Similar to SiamBAN^3^, we do not search for the hyper-parameters of the loss function and simply set $\lambda _1=\lambda _2=1$.

## Experiments

### Implementation details

Our approach is implemented in Python using Pytorch on a PC with an Intel i7 CPU and four NVIDIA GeForce 1080Ti GPU.

**Table 1:** Performance comparisons on VOT-2018. italic, bolditalic and underline fonts indicate the top-3 trackers.

VOT-2018	SiamFC++	PrDiMP	SiamAttn	SiamRPNpp	DiMP	SiamBAN	*SiamFDA*
EAO ($\uparrow$)	0.426	0.442	${\varvec{0.47}}$	0.417	0.441	0.452	${\textit{0.476}}$
Accuracy ($\uparrow$)	0.587	${\varvec{0.618}}$	$\textit{0.63}$	0.604	0.597	0.597	0.598
Robustness ($\downarrow$)	0.183	0.165	$\underline{0.16}$	0.234	${\varvec{0.152}}$	0.178	0.178

#### Training phase

 Our proposed SiamFDA is trained end-to-end with image pairs picked from ImageNet VID^21^, YouTube BoundingBoxes^22^, COCO^23^, ImageNet DET^21^, GOT10k^24^ and LaSOT^25^, using Stochastic Gradient Descent(SGD) with a minibatch of 32 pairs. The size of an template patch is $127\times {127}$ pixels, and the size of a search patch is $225\times {225}$ pixels. We adopt the modified ResNet-50^3^ pretrained from ImageNet^21^ as the backbone and the parameters of the first two layers are frozen. The total training epoch is 20. We first train our model for 5 warm up epochs with a learning rate linearly increased from 0.001 to 0.005, then use a learning rate exponentially decayed from 0.005 to 0.00005 in the last 15 epoches. In the first 10 epochs, we only train those layers without pretraining, and fine-tune the remaining parameters in the last 10 epochs.

#### Tracking phase

The template feature in the first frame is computed via the Siamese backbone once, and then is continuously matched to subsequent search images, generating the target center location and bounding boxes via the classification branch and regression branch, respectively. In order to achieve a more stable and smoother prediction between adjacent frames, cosine windows and scale change penalties^2^ are used. Cosine windows reduce boundary effects by applying a cosine-shaped weight distribution within the tracking window, placing the highest weight at the center and gradually decreasing towards the edges. This method focuses on the target at the center of the window, minimizing the disruptive influence of the window’s edges, thereby making the tracking process smoother and more focused. On the other hand, scale change penalties are employed to manage changes in the target’s size within the video. As the target moves away from or closer to the camera, its size in the frame changes. By penalizing rapid or significant scale changes, this mechanism assists the tracking algorithm in smoothly and gradually adjusting the size of the tracking window, avoiding instability due to abrupt scale changes. The combination of these two techniques significantly enhances the coherence and stability of frame-to-frame predictions, improving the overall efficacy of the tracking algorithm. Then, we identify the predicting bounding box with the highest score as the most probable location of the target in each frame. This bounding box is then linearly interpolated with the states from historical frames to maintain a continuous and accurate trajectory of the target. This interpolation not only utilizes the current frame’s data but also leverages the historical information, ensuring a more reliable tracking even when the target undergoes sudden changes in motion or appearance. Subsequently, the target state is updated based on this interpolated data, which includes the target’s updated position and size. To further enhance tracking accuracy, especially in scenarios of occlusion where the target is partially or completely obscured, we employ a Kalman filter. This filter assists in predicting the target’s location by extrapolating from previous observations, thereby compensating for moments when the target is not clearly visible. The integration of a Kalman filter proves crucial in maintaining robust tracking in complex environments, effectively mitigating the challenges posed by occlusions.

**Figure 6:**
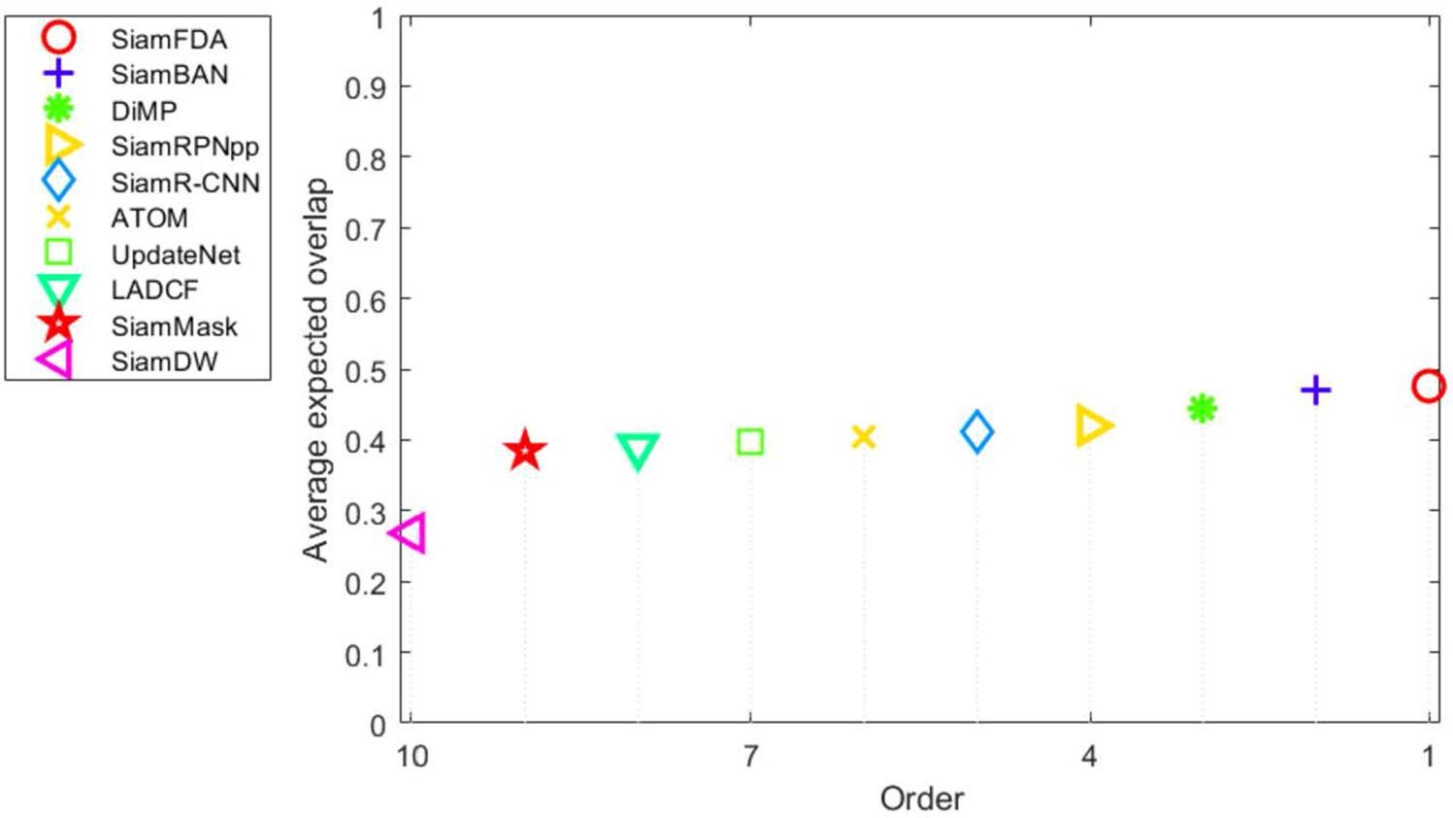
Expected average overlap (EAO) graph with trackers ranked from right to left on VOT-2018. The right-most tracker achieves the top-performing result.

### Comparison with the state-of-the-arts

Six datasets including VOT-2018^26^, VOT-2019^27^, GOT10k^24^, LaSOT^25^, OTB-2015^28^ and OTB-2013^29^ are adopted to demonstrate the performance of our SiamFDA tracker against numerous SOTA trackers.

**Table 2:** Performance comparisons on VOT-2019. italic, bolditalic and underline fonts indicate the top-3 trackers.

VOT-2019	SiamDW	ATOM	DCFST	SiamRPNpp	DiMP	SiamBAN	*SiamFDA*
EAO ($\uparrow$)	0.299	0.301	0.317	0.285	$\underline{0.321}$	${\varvec{0.327}}$	${\textit{0.351}}$
Accuracy ($\uparrow$)	$\underline{0.6}$	$\textit{0.603}$	0.585	0.599	0.582	${\varvec{0.602}}$	0.599
Robustness ($\downarrow$)	0.467	0.411	$\underline{0.376}$	0.482	${\varvec{0.371}}$	0.396	${\textit{0.356}}$

**Table 3:** Performance comparisons on GOT10k. italic, bolditalic and underline fonts indicate the top-3 trackers.

GOT10k	SiamMASK	DaSiamRPN	SiamFC++	SiamRPNpp	DiMP	*SiamFDA*
AO ($\uparrow$)	0.514	0.444	$\underline{0.595}$	0.518	${\varvec{0.611}}$	${\textit{0.615}}$
SR_0.5_ ($\uparrow$)	0.587	0.536	$\underline{0.695}$	0.618	${\varvec{0.717}}$	${\textit{0.731}}$
SR_0.75_ ($\uparrow$)	0.366	0.22	${\varvec{0.479}}$	0.329	$\textit{0.492}$	${\underline{0.477}}$

**Figure 7:**
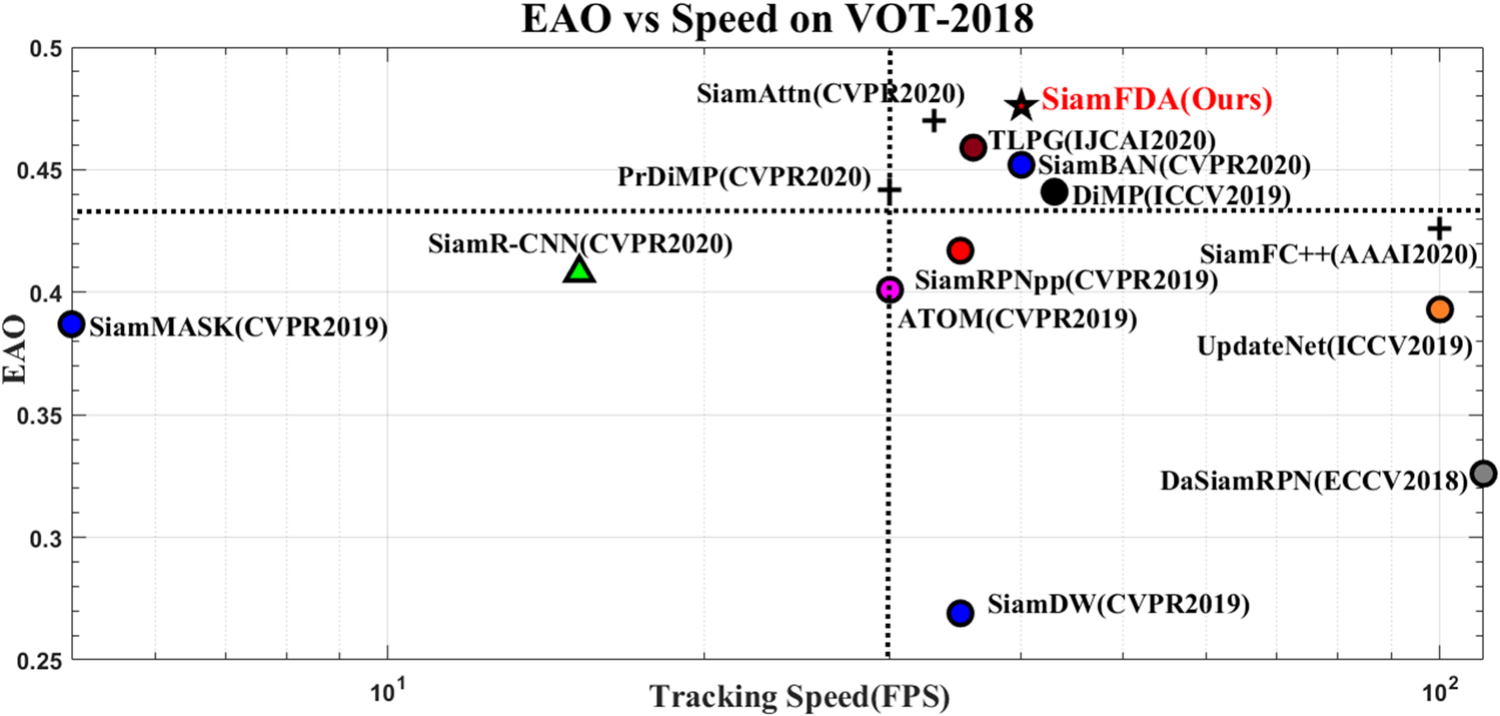
A comparison of the quality and the speed of SOTA trackers on VOT-2018.

#### VOT-2018

VOT-2018^26^ contains 60 sequences and adopts expected average overlap (EAO) as the major evaluation metric, which measures robustness (failure rate) and accuracy (average overlap). We compare our tracker with several SOTA trackers, including SiamFC++^11^, PrDiMP^30^, TLPG^31^, SiamAttn^20^, ATOM^32^, SiamR-CNN^33^, SiamRPNpp^9^, DiMP^34^, SiamBAN^3^, UpdateNet^35^, LADCF^36^, SiamMASK^37^ and SiamDW^38^. Table 1 and Fig. 6 show that, compared with almost all the top-performing trackers in VOT2018, our SiamFDA tracker achieves the best EAO score of 0.476. Besides, we also visualize EAO with respect to the tracking speed, as Fig. 7 shown. From the plot, our SiamFDA achieves best performance, while still running at real-time speed (40 fps).

**Figure 8:**
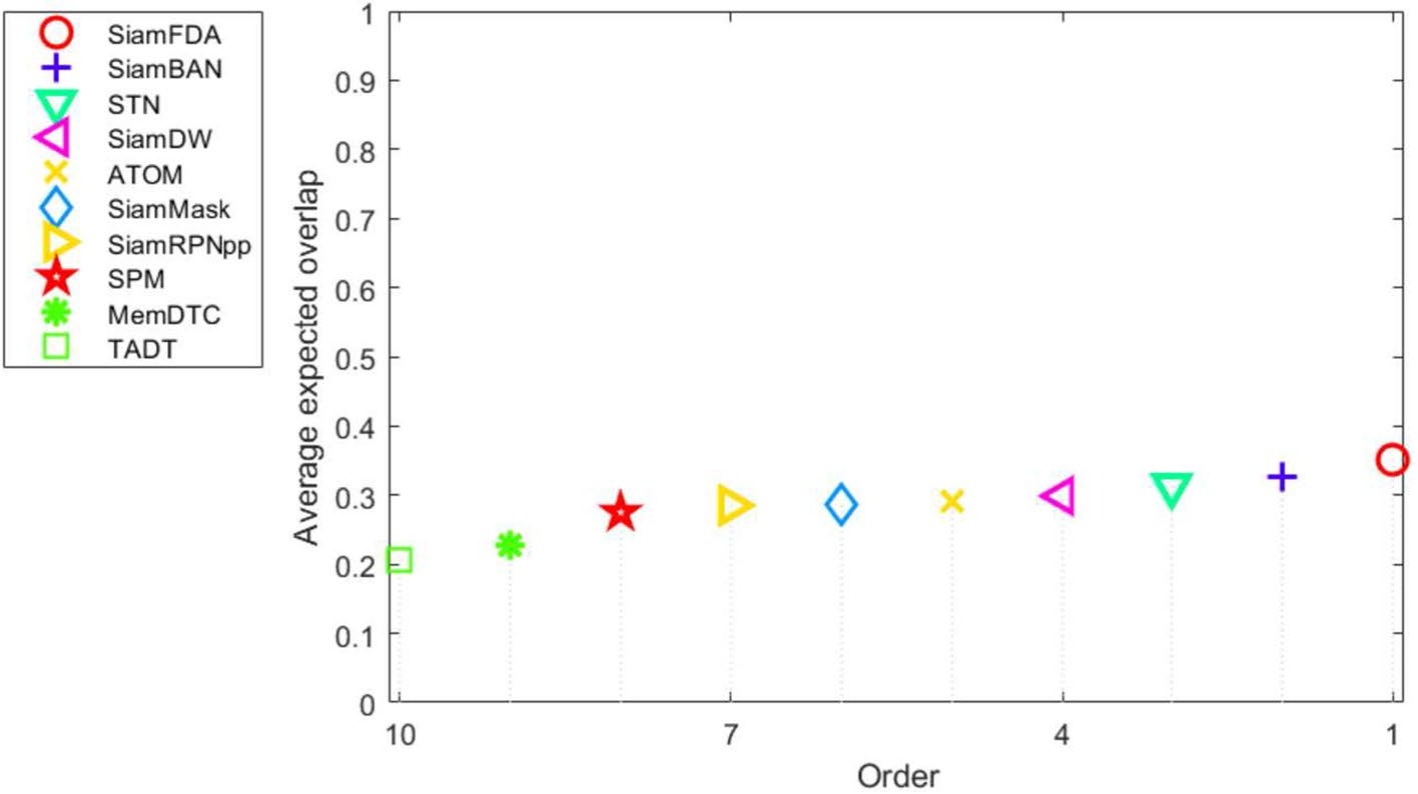
Expected average overlap (EAO) graph with trackers ranked from right to left on VOT-2019. The right-most tracker achieves the top-performing result.

#### VOT-2019

VOT-2019^27^ replaces $20\%$ easy sequences of VOT-2018^26^. We compared our tracker with recent prevailing trackers, including SiamDW^38^, SiamMask^37^, ATOM^32^, DCFST^27^, SiamRPNpp^9^, DiMP^34^, SiamBAN^3^, STN^39^, SPM^40^, MemDTC^41^ and TADT^42^. Table 2 and Fig. 8 show that our SiamFDA tracker has the highest EAO and obtains 2.4% relative increases over SiamBAN^3^. It is worth noting that the improvement of our SiamFDA mainly comes from the robustness score, which outperforms SiamBAN^3^ by 4%.

#### GOT10k

GOT10k^24^ test set is a large-scale high-diversity dataset, containing 180 videos, with the average overlap (AO) and success rates (SR) at two thresholds as measure metrics. We evaluate our SiamFDA with SiamFC^1^, DaSiamRPN^43^, SiamMask^37^, ATOM^32^, SiamFC++^11^, SiamRPNpp^9^ and DiMP^34^. Results on GOT10k are reported in Table 3, from which, we can conclude that our SiamFDA significantly outperforms nearly all top-performing SOTA trackers in all performance metrics.

#### LaSOT

**Figure 9:**
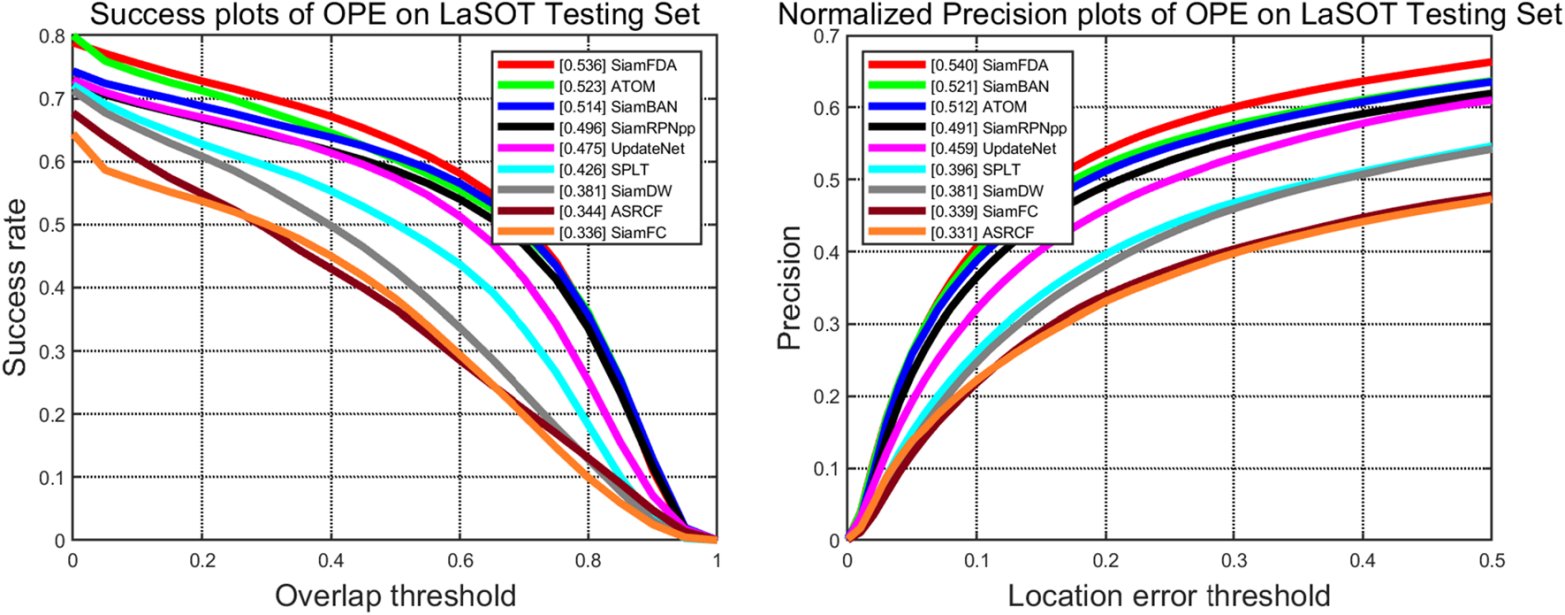
Success and normalized precision plots on LaSOT.

LaSOT^25^ test set (280 videos, average length of 2448 frames) is a long-term visual object tracking evaluation dataset, which uses success plots and normalized precision plots to evaluate tracking performance. We evaluate our tracker with trackers including SiamBAN^3^, SiamRPNpp^9^, UpdateNet^35^, SPLT^44^, SiamDW^38^, ASRCF^45^, ATOM^32^ and SiamFC^1^. Figure 9 shows that our SiamFDA tracker achieves an advantageous result with a success rate of 0.536 and 0.540 normalized precision.

#### OTB-2015

**Figure 10:**
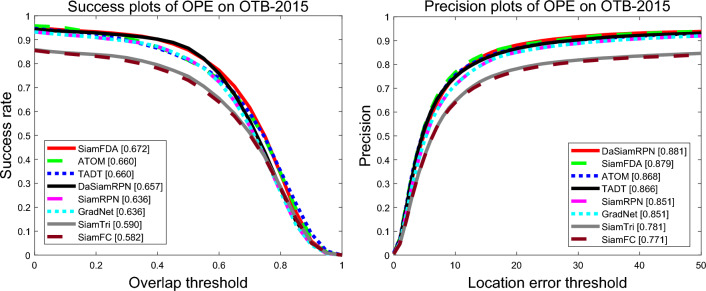
Success and normalized precision plots on OTB100.

OTB-2015^28^ consists of 100 sequences and adopts one-pass evaluation (OPE) success plots and precision plots as evaluation metrics. Our SiamFDA tracker is compared with numerous SOTA trackers including ATOM^32^, TADT^46^, DaSiamRPN^43^, SiamRPN^2^, GradNet^47^, SiamTri^48^ and SiamFC^1^. As results displayed in Fig. 10, our SiamFDA tracker is dominant over other trackers, with a success score of 0.672 and a precision score of 0.879.

#### OTB-2013

OTB-2013^29^ consits of 50 challenging image sequences, which is a subset of OTB-2015^28^ and annotated with bounding boxes with several different attributes. Besides, we compare our tracker SiamFDA with other SOTA trackers including TADT^46^, SiamRPN^2^, GradNet^47^, DaSiamRPN^43^, ATOM^32^, SiamTri^48^ and SiamFC^1^. Table 4 shows that that our proposed SiamFDA performs favorably against other outstanding trackers especially when encountering with low resolution and background clutter.

**Table 4:** Comparisons on OTB-50, evaluated by precision and success rate.

Method	Low resolution		Background clutter	
	Precision	Success rate	Precision	Success rate
TADT^46^	$\underline{0.875}$	0.680	$\underline{0.875}$	0.680
SiamRPN^2^	0.789	0.601	0.789	0.601
DaSiamRPN^43^	0.872	0.667	0.872	0.667
ATOM^32^	0.810	0.621	0.810	0.621
SiamTri^48^	${\varvec{0.884}}$	${\varvec{0.692}}$	${\varvec{0.884}}$	${\varvec{0.692}}$
SiamFC^1^	0.749	0.573	0.749	0.573
$\textbf{SiamFDA}$	$\textit{0.889}$	$\textit{0.701}$	$\textit{0.889}$	$\textit{0.701}$

Italic, bolditalic, and underline fonts indicate the top-3 trackers.

#### TNL2K

TNL2K represents a recently developed benchmark specifically tailored for visual-language (VL) tracking, encompassing a comprehensive dataset with 2000 video sequences. This benchmark distinguishes itself through a combination of key attributes, including superior quality, the inclusion of challenging adversarial samples, and extensive variation in appearance. We compare our tracker SiamFDA with other SOTA trackers including TNL2K^49^, SNLT^50^, CTRNLT^51^, VLTTT^52^, JointNLT^53^. Table 5, from which, We can conclude that SiamFDA exhibits superior performance on the assessed dataset compared to most of the current state-of-the-art methods. Notably, even though Transformer-based approaches surpass SiamFDA in accuracy, they significantly fall short in terms of real-time performance. This juxtaposition highlights SiamFDA’s advantage in delivering efficient tracking capabilities, particularly in scenarios that demand rapid response and minimal computational resources. Therefore, despite the superior accuracy of Transformer-based methods, SiamFDA emerges as a more practical solution for real-time tracking, striking a balance between high accuracy and operational feasibility.

**Table 5:** Performance comparisons on TNL2K.

TNL2K	CTRNLT	VLTTT	JointNLT	TNL2K-2	SNLT	*SiamFDA*
SUC ($\uparrow$)	0.44	$\underline{0.531}$	$\textit{0.569}$	0.42	0.276	${{\varvec{0.542}}}$
Norm.PRE_0.5_ ($\uparrow$)	0.52	${\varvec{0.593}}$	$\textit{0.796}$	0.50	–	${\underline{0.572}}$
PRE_0.75_ ($\uparrow$)	0.45	${\varvec{0.533}}$	$\textit{0.581}$	0.42	0.419	${\underline{0.528}}$

Italic, bolditalic and underline fonts indicate the top-3 trackers.

### Ablation studies

**Table 6:** Ablation studies of SiamFDA on VOT-2018 and VOT-2019.

Component	A1	A2	A3	A4	A5
FDA block			$\surd$		$\surd$
NL block				$\surd$	
SE block		$\surd$	$\surd$	$\surd$	$\surd$
Ellipse					$\surd$
Circle	$\surd$	$\surd$	$\surd$	$\surd$	
EAO in VOT-2018	0.406	0.435	0.476	0.430	0.447
EAO in VOT-2019	0.281	0.314	0.351	0.309	0.323

Ablation studies are performed on VOT-2018^26^ and VOT-2019^27^ to demonstrate the impact of key components of SiamFDA. As shown as Table 6, FDA block, NL block, SE block represent feature dynamic activation block, non-local block and squeeze-and-excitation block. Rectangle, Circle represent rectangle labels ($E1+E2$), adaptive labels ($E1+E2/C2$), respectively.

#### Ablation studies on blocks

As shown as Table 6, we perform an ablation study on the effects of blocks we adopt. Compared *A*1 with *A*2, we can found that the introduction of SE block makes the EAO criterion increases from 0.406 to 0.435 on VOT-2018^26^ and 0.281 to 0.314 on VOT-2019^27^. Based on *A*2, when using our proposed FDA block, the performance achieves better results. From *A*3 to *A*4, though NL blocks^5^ reach competitive results on object detection/segmentation, it’s not effective enough when applied directly to object tracking, and we speculate that this is because of the essential difference among these fields.

#### Ablation studies on sample label assignments

To explore the impact of sample label assignments on tracking performance, we take the target shape into account. Compared *A*3 with *A*5, we can reach a conclusion that the adaptive sample label assignment method contributes to better tracking results.

## Conclusions

In this paper, we propose a novel anchor-free network named SiamFDA, which consists of a Siamese network backbone for feature extraction and a feature dynamic activation subnetwork for accurate target location estimation as well as bounding box prediction. Specifically, a simple yet effective FDA block is designed to capture long-range dependencies between distant pixels in space and further activate reliable regions, thus improving the tracking robustness. Besides, a SE block serves as a hierarchical feature selector to focus on features which are more advantageous to track the current target. Furthermore, we adjust the sample label assignment method adaptively according to the target shape. Extensive experiments are conducted on five datasets, where our method obtains competitive results, with real-time running speed.

## Data Availability

The datasets generated during and/or analysed during the current study are available from the corresponding author on reasonable request.
